# Prolonged growth hormone pretreatment is associated with improved embryo quality in women with poor quality embryos in previous IVF cycles

**DOI:** 10.1186/s12884-026-09825-4

**Published:** 2026-08-15

**Authors:** Chang Liu, Peiyu Wang, Peipei Pan, Haiyan Yang, Xuefeng Huang

**Affiliations:** Reproductive Medicine Center, The First Affiliated Hospital of Wenzhou Medical University, Wenzhou, Zhejiang 325000 People’s Republic of China

**Keywords:** Poor embryo quality, Growth hormone, In vitro fertilization

## Abstract

**Background:**

Approximately 10–15% of in vitro fertilization (IVF) and intracytoplasmic sperm injection (ICSI) cycles have most embryos of poor quality, often characterized by extensive anuclear cytoplasmic fragments. These cycles are usually associated with poor reproductive outcomes, including diminished implantation, clinical pregnancy, and live birth chance. Growth hormone (GH) has emerged as a potential adjuvant therapy to improve oocyte and embryo quality in these IVF cycles. However, the optimal duration of GH treatment in women with poor quality embryos remains unclear.

**Objective:**

To evaluate whether prolonged GH pretreatment is associated with better IVF outcomes in women with poor quality embryos in previous cycles.

**Methods:**

A retrospective cohort study was conducted at the Reproductive Center of the First Affiliated Hospital of Wenzhou Medical University, including women with poor embryo quality in previous IVF cycles. Patients were divided into three groups based on GH pretreatment duration: non-GH (Group A), 2–4 weeks GH (Group B), and 12 weeks GH (Group C). IVF outcomes, including D3 good-quality embryo rate, blastocyst formation rate, and fresh and cumulative live birth rates, were compared. Statistical analyses were performed using Kruskal–Wallis rank-sum tests and multivariate linear regression.

**Results:**

Group C had significantly higher D3 good-quality embryo rate (46.17% ± 33.57%) compared with Group A (25.66% ± 25.41%, *P* < 0.001) and Group B (30.93% ± 25.45%, *P* = 0.02). Multivariate regression analysis confirmed that GH pretreatment for 12 weeks had significantly increased D3 embryo quality when compared with GH pretreatment for 2–4 weeks and no GH pretreatment. Additionally, Group B and C exhibited significantly higher blastocyst formation rates compared with Group A. Regarding the clinical outcomes, Group C achieved a significantly higher live birth rate following fresh embryo transfer (57.14%) than Group A (25.64%) (*P* = 0.04), but had non significant higher cumulative live birth rate than group A and B.

**Conclusion:**

Prolonged GH pretreatment for 12 weeks was associated with improved embryo quality in women with poor embryo quality in previous IVF cycles. Although a higher live birth rate was observed in fresh embryo transfer cycles, no significant improvement was found in cumulative live birth rate.

**Supplementary Information:**

The online version contains supplementary material available at https://doi.org/10.1186/s12884-026-09825-4.

In women undergoing IVF/ICSI (in vitro fertilization/intracytoplasmic sperm injection) treatment, poor quality embryo, mainly characterized by extensive anuclear cytoplasmic fragments in its cleavage stage, may consist up to more than 70–80% total embryos in approximately 10–15% of the cycles. The degree of fragmentation has independent prognostic power to predict live birth and comprises as a part of the standardized morphological grading system, which provides a reference for embryologists in the selection of embryos for transfer during an IVF/ICSI cycle [1, 2]. Moreover, compelling evidence shows that the transfer of embryos with lower quality rated by embryonic grading system has been associated with unfavorable reproductive outcomes, including diminished implantation, clinical pregnancy and live birth chance [3]. However, women with poor quality embryos do not necessarily share similar clinical characteristics and usually cannot be predicted before initiation of IVF cycle. They tend to undergo repetitive cycles with poor quality embryos even after the application of various ovarian stimulation protocols [4]. The primary cause of poor-quality embryos during the early stage remains elusive, but oocyte dysfunctions, including mitochondrial dysfunction, apoptosis, programmed cell death, telomere attrition, free radical production, meiotic spindle dysfunction and genomic instability, are considered as the leading contributors [5–7]. Therefore, improving oocyte quality is one of the key targets to improve embryo quality. In recent years, multiple adjuvant therapies have been used to improve embryo quality, such as the pretreatment with coenzyme Q10, inositol, and growth hormone [8–10].

Growth hormone (GH), primarily produced by the pituitary gland, also functions as an autocrine and paracrine regulator of female reproductive processes. This is attributed to its local synthesis in the ovaries and uterus, along with the presence of GH receptors in granulosa cells, theca cells, oocytes, and the endometrial tissue. The expression of GH helps increase the production of insulin-like growth factor 1 (IGF-1) in the ovaries, which is critical for the formation of luteinizing hormone receptors, aromatase activity, oocyte maturation, and the production of estrogen and progesterone [11]. It has been reported that the concentration of GH in follicular fluid is associated with embryo quality [10]. GH has been widely applied in women at advanced age or with diminished ovarian reserve when undergoing IVF treatment [12, 13]. Clinical studies evaluating the role of GH in IVF cycle have shown inconsistent results [14–16]. Currently, most clinical applications of GH in IVF have a treatment duration of less than 1 month [17–19]. Moreover, the application of GH in women with poor quality embryos in previous IVF cycles has rarely been reported. To the best of our knowledge, the development of follicles from the primordial follicle stage to the pre-ovulatory mature follicle typically takes about 85 days [20]. Clinical research on the impact of extending GH treatment duration on IVF outcomes is insufficient. In view of this evidence gap, we conducted this study aiming to explore whether prolonged pretreatment duration of GH can result in better reproductive outcomes in women with poor-quality embryos.

## Materials and methods

### Study design

**Fig. 1:**
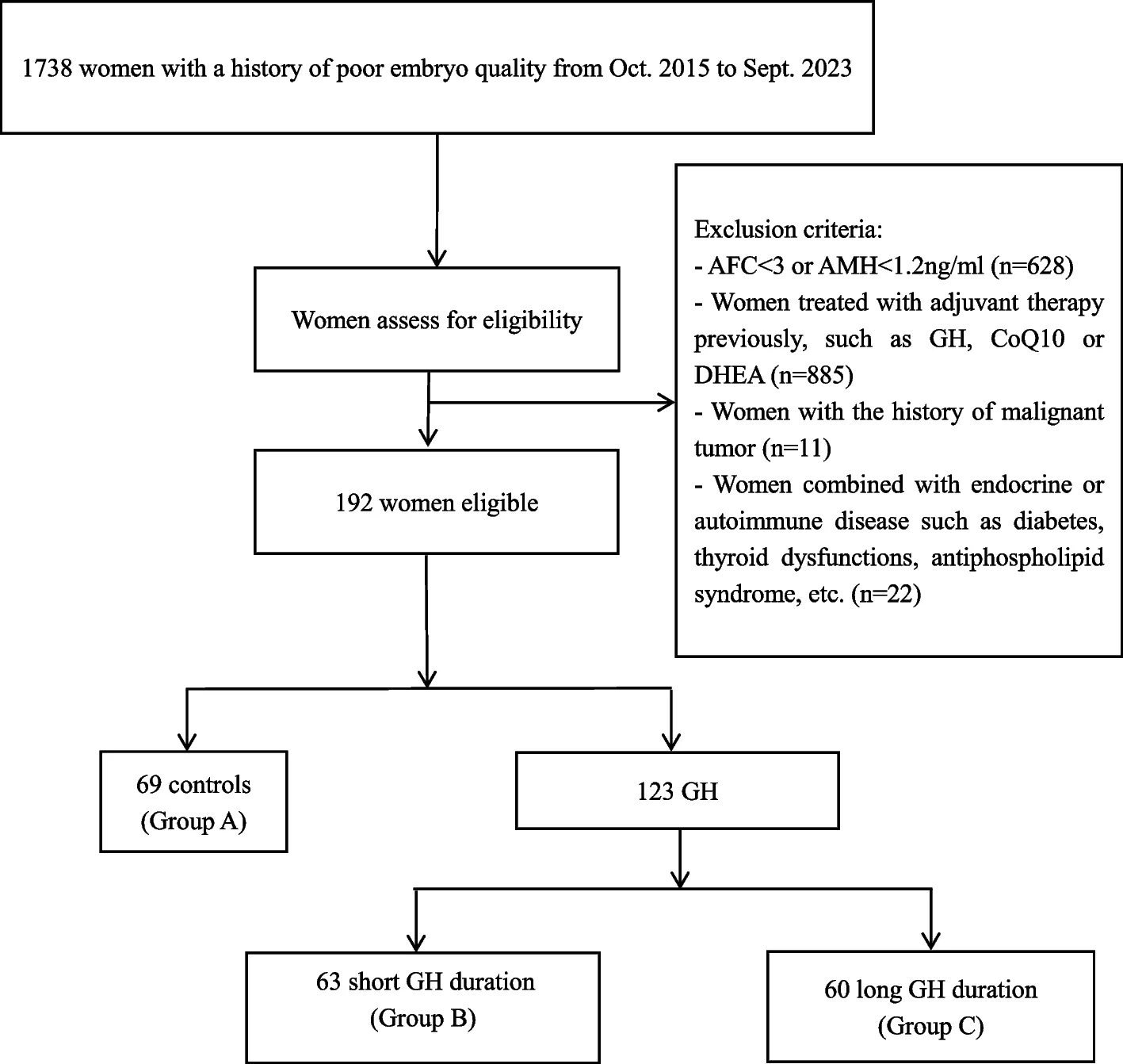
Flow chart of study design

A retrospective, single-centered cohort study was conducted at the Reproductive Center of the First Affiliated Hospital of Wenzhou Medical University. A total of 1738 women with a previous IVF cycle of poor embryo quality between October 2015 and September 2023 were included in the study. The inclusion criteria were (1) women ≤ 38 years of age; (2) the proportion of D3 high-quality embryos (embryo fragmentation < 20% and > 6 cells) among all Day 3 (D3) embryos was < 20% in an IVF/ICSI cycle; (3) no GH was used in any previous cycles. The exclusion criteria included (1) antral follicle count (AFC) < 3 or anti-Müllerian hormone (AMH) < 1.2 ng/mL; (2) women treated with adjuvant therapy previously, such as CoQ10 or DHEA; (3) women with the history of malignant tumor; (4) women combined with endocrine or autoimmune disease such as diabetes, thyroid dysfunctions, antiphospholipid syndrome, etc. Ultimately, a total of 192 women were included in final analysis. The flow chart of the study was illustrated in Fig. 1. This study was approved by the institutional review board of the First Affiliate Hospital of Wenzhou Medical University (No.KY2025-R021). All methods were performed in accordance with the relevant guidelines and regulations, and in accordance with the Declaration of Helsinki. Written informed consent was obtained for the whole procedure from all the couples.

### Ovarian stimulation protocols

The ovarian stimulation protocols in this study were classified into three categories, including GnRH antagonist protocol, GnRH agonist protocol and progestin-primed ovarian stimulation (PPOS) protocol. There were no different indications for these three ovarian stimulation protocols. Each protocol was applied according to the overall clinical circumstances and the discretion of the clinicians. In the GnRH agonist long protocol, pituitary suppression was achieved with a single intramuscular injection of Triptorelin (3.75 mg, Ferring Pharmaceuticals, France), followed by stimulation with recombinant FSH (r-FSH, Serono, Switzerland) at a starting dose of 150–250 IU, which was adjusted according to ovarian response. In the GnRH antagonist protocol, Cetrorelix Acetate (0.25 mg, Serono, Switzerland) was administered daily starting on the 5th or 6th day of r-FSH stimulation. In the progestin-primed protocol, medroxyprogesterone acetate (MPA, 10 mg, Livzon Pharm, China) was combined with HMG (Livzon Pharm, China) and administered daily, while r-FSH or HMG was initiated at a starting dose of 150–250 IU and adjusted based on ovarian response. The patients were provided with 5000–10,000 IU of hCG when the leading follicle reached 18 mm in diameter with at least three follicles > 16 mm in diameter. The oocytes were collected 36 h after hCG trigger. No more than two embryos were transferred after oocyte retrieval. Luteal support was provided through oral administration of dydrogesterone (20 mg daily) and vaginal progesterone (90 mg daily), beginning on the day of oocyte retrieval for two weeks, and, if pregnant, continuing until the 8th week of gestation. Surplus embryos were cryopreserved by vitrification at the cleavage stage (day 3) or blastocyst stage (day 5/6). For patients undergoing frozen embryo transfer (FET), hormone replacement treatment was used for endometrial preparation and were detailed in our previous study [21].

### Growth hormone supplementation

Based on the use of growth hormone and the duration of its administration until oocyte retrieval, patients were divided into three groups: Group A (no GH use before or during this IVF cycle), Group B (GH pre-treatment for 2–4 weeks), and Group C (GH pre-treatment for 12 weeks). Recombinant human GH (Jintropin; GenSci, China) was administered subcutaneously at a dose of 4.5 IU every other day prior to the initiation of ovarian stimulation, followed by a daily subcutaneous injection of 4.5 IU after the initiation of controlled ovarian stimulation, continuing until oocyte retrieval.

### Embryo quality assessment

D3 embryo quality was assessed at 72 h after oocyte retrieval. Embryos were rated according to a combination of blastomere number, blastomere size and fragmentation. Embryos with > 6 blastomeres, equal or slightly unequal size and ≤ 25% anucleate fragments were regarded as good quality embryos. For women with more than two good quality embryos in day 3, two good quality embryos were transferred or frozen for future use. The remaining embryos were cultured to the blastocyst stage. The quality of blastocysts was outlined according to Gardner and Schoolcraft's classification [22]. Specifically, AA blastocysts are characterized by good quality inner cell mass and trophectoderm, AB by good quality inner cell mass and average quality trophectoderm, BA by average quality inner cell mass and good quality trophectoderm, BB by average quality inner cell mass and trophectoderm. Blastocysts rated as 3BB and higher grade (i.e., AA, AB, BA and BB) are classified as good quality blastocysts.

### Outcome definitions

The primary outcomes of the study were the D3 good quality embryo rate, defined as the number of D3 good quality embryos divided by the total number of cleaved embryos. Other outcomes included the total Gn dosage and duration, number of oocytes retrieved, number of MII oocytes, blastocyst formation rate, clinical pregnancy rate and live birth rate following fresh embryo transfer. The clinical pregnancy was defined as the presence of a gestational sac with fetal heart activity under ultrasound examination 35 days after embryo transfer[23]. A live birth was defined as baby born after 22 weeks of gestational age [23].

### Statistical analysis

R4.3.2 software was used for all statistical analyses in the study. Normally distributed continuous variables were assessed for normality using the One-sample Kolmogorov–Smirnov test and were presented as mean and standard deviation; otherwise, they were presented as median (interquartile range). The qualitative data were expressed as percentages. Differences between groups were analyzed using the Kruskal–Wallis rank-sum test for continuous variables and Fisher’s exact test for categorical variables. Univariate analyses were performed to examine the relationships between relevant parameters and the D3 good-quality embryo rate. For multivariable analysis, linear or logistic regression models were used to assess the independent effects of predictors on the primary and secondary outcomes. Variables with a *p*-value of less than 0.2 in univariate analysis were included in the multivariable regression models. All *p*-values were two-tailed, and *p* < 0.05 was considered statistically significant.

## Results

### Baseline characteristics of women in three groups

The baseline characteristics of women across three groups were presented in Table 1, with no significant differences in age, infertility duration, primary infertility proportion, body mass index (BMI), AMH levels, AFC, basal follicle stimulation hormone (FSH), basal luteinizing hormone (LH), or basal estradiol (E2) levels (all *P* > 0.05).

**Table 1:** Baseline characteristics in three groups

	Group A (*n* = 69)	Group B (*n* = 63)	Group C (*n* = 60)	*P*-value
Age (year)	30.48 ± 3.25	31.10 ± 2.88	31.63 ± 3.40	0.169
Infertility duration (year)	3.96 ± 2.64	3.84 ± 2.69	3.49 ± 2.62	0.497
Primary infertility (n, %)	37 (53.62%)	26 (41.27%)	22 (36.67%)	0.128
BMI (kg/m^2^)	21.62 ± 3.36	21.46 ± 2.49	22.21 ± 3.80	0.499
AMH (ng/mL)	3.06 ± 2.28	4.52 ± 3.65	3.61 ± 3.80	0.356
AFC (n)	17.19 ± 8.62	14.76 ± 8.32	18.28 ± 11.39	0.116
Basal FSH (IU/L)	7.85 ± 2.32	8.04 ± 2.55	7.80 ± 2.24	0.976
Basal LH (IU/L)	4.93 ± 2.06	5.51 ± 3.88	5.28 ± 3.62	0.774
Basal E2 (pmol/L)	187.57 ± 86.22	187.68 ± 74.50	167.77 ± 93.14	0.073

*BMI* body mass index, *AMH* anti-mullerian hormone, *AFC* antral follicle count, *FSH* follicle stimulating hormone, *LH* luteinizing hormone, *E2* estradiol

### IVF treatment and embryonic outcomes in three groups

The IVF treatment and embryonic outcomes among the three groups were shown in Table 2. Significant differences were observed in the ovarian stimulation protocols (*P* < 0.001), with Group A predominantly using the GnRH agonist protocol (81.16%), while Group C had a higher proportion of patients receiving the GnRH antagonist protocol (45.00%) and PPOS (31.67%). Gonadotropin (Gn) duration also differed significantly among the groups (*P* = 0.01), with Group C requiring a shorter duration compared to Groups A and B.

**Table 2:** IVF treatment and embryonic outcomes in three groups

	Group A (*n* = 69)	Group B (*n* = 63)	Group C (*n* = 60)	*P*-value
Ovarian stimulation protocol (n, %)				< 0.001^bc^
GnRH agonist	56 (81.16%)	43 (68.25%)	14 (23.33%)	
GnRH antagonist	10 (14.49%)	10 (15.87%)	27 (45.00%)	
PPOS	3 (4.35%)	10 (15.87%)	19 (31.67%)	
Fertilization type (n, %)				0.291
IVF	29 (42.03%)	36 (57.14%)	28 (46.67%)	
ICSI	39 (56.52%)	25 (39.68%)	29 (48.33%)	
IVF/ICSI	1 (1.45%)	2 (3.17%)	3 (5.00%)	
Gn duration (day)	11.13 ± 2.07	11.24 ± 3.37	9.97 ± 2.48	0.010^bc^
Gn dosage (IU)	2,238.41 ± 983.52	2,691.23 ± 1,510.98	2,237.09 ± 1,239.02	0.150
No. of retrieved oocytes (n)	14.26 ± 8.39	12.10 ± 6.44	12.28 ± 8.62	0.233
No. of mature oocytes (n)	11.45 ± 6.77	10.37 ± 5.70	9.82 ± 7.49	0.174
No. of D3 good quality embryos (n)	2.00 ± 2.43	2.71 ± 2.95	3.19 ± 4.43	0.130
D3 good quality embryo rate (%)	25.66 ± 25.41	30.93 ± 25.45	46.17 ± 33.57	0.001^bc^
No. of blastocysts (n)	0.39 ± 1.15	1.22 ± 2.11	1.34 ± 2.30	0.010^ab^
Blastocyst formation rate (%)	3.95 ± 10.28	15.57 ± 23.78	17.82 ± 24.82	0.005^ab^
Good-quality blastocyst formation rate (%)	1.59 ± 5.09	3.50 ± 11.03	3.27 ± 8.47	0.802

*IVF* in vitro fertilization, *GnRH* gonadotropin-releasing hormone, *PPOS* progestin-primed ovarian stimulation, *ICSI* Intracytoplasmic sperm injection, *Gn* gonadotropin

^a^Group A vs. Group B, *P* < 0.05

^b^Group A vs. Group C, *P* < 0.05

^c^Group B vs. Group C, *P* < 0.05

Regarding embryonic outcomes, Group C exhibited the highest D3 good-quality embryo rate (46.17% ± 33.57%), which was significantly higher than that of Groups A (25.66% ± 25.41%) and B (30.93% ± 25.45%) (*P* = 0.001). The number of blastocysts and blastocyst formation rates were significantly higher in Groups B and C compared to Group A (*P* = 0.010 and *P* = 0.005, respectively). However, there were no significant differences in fertilization type, Gn dosage, the number of retrieved or mature oocytes, or good-quality blastocyst formation rate among the groups (all *P* > 0.05).

### Stratified analysis according to ovarian stimulation protocols

**Fig. 2:**
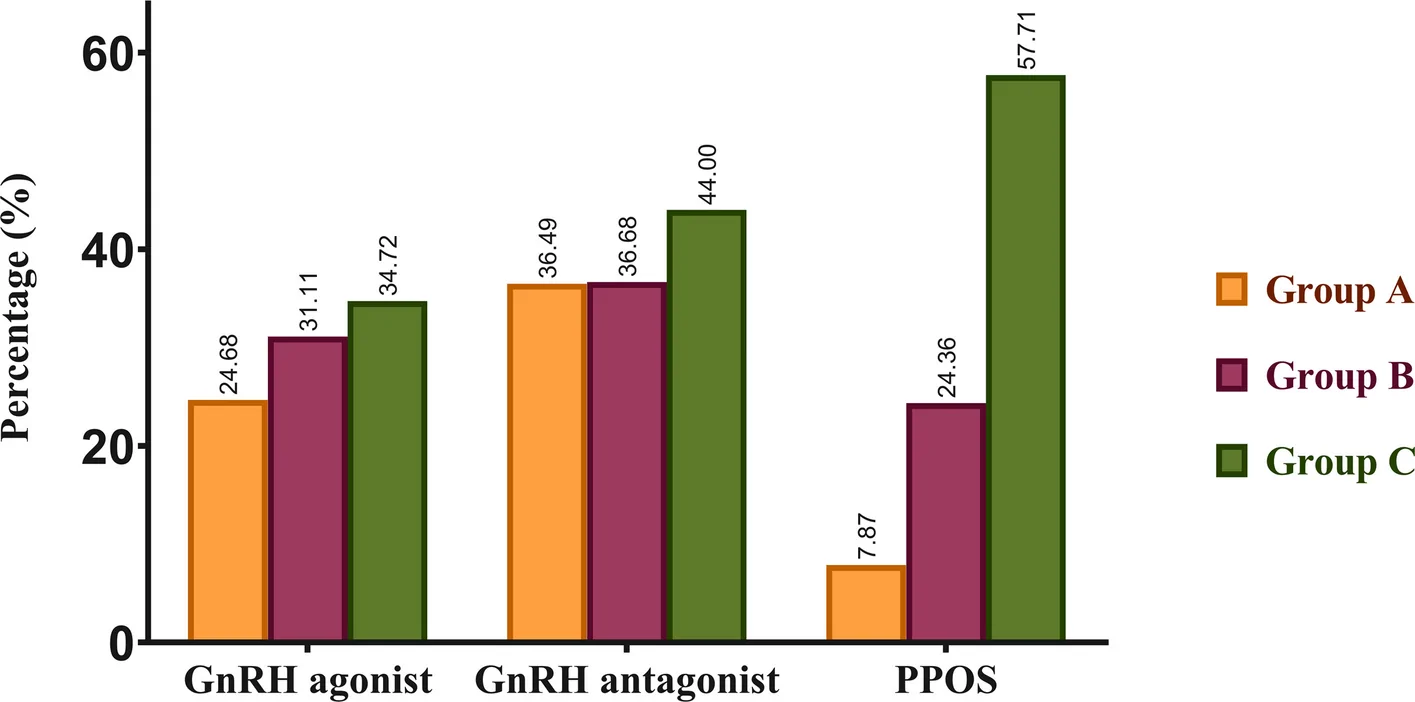
D3 good quality embryo rate in three groups stratified by ovarian stimulation protocols. Note: The results in the PPOS subgroup should be interpreted with caution due to the small size

Given the significant differences in ovarian stimulation protocols among the three groups, stratified analyses were performed according to ovarian stimulation protocols (Fig. 2). Across all stimulation protocol subgroups, a consistent trend toward higher D3 good-quality embryo rates was observed with prolonged GH pretreatment (Group C) compared with Groups A and B. In the GnRH agonist subgroup, the D3 good-quality embryo rate increased from 24.68% in Group A to 31.11% in Group B and 34.72% in Group C. Similarly, in the GnRH antagonist subgroup, the corresponding rates were 36.49%, 36.68%, and 44.00%, respectively. In the PPOS subgroup, a higher D3 good-quality embryo rate was also observed in Group C (57.71%) compared with Group B (24.36%) and Group A (7.87%). However, this finding should be interpreted with caution due to the very small sample size in Group A (*n* = 3). Although the analyses in GnRH agonist and GnRH antagonist subgroup did not reach statistical significance, likely due to limited sample sizes, no contradictory patterns were observed across subgroups.

### Clinical outcomes in three groups

**Fig. 3:**
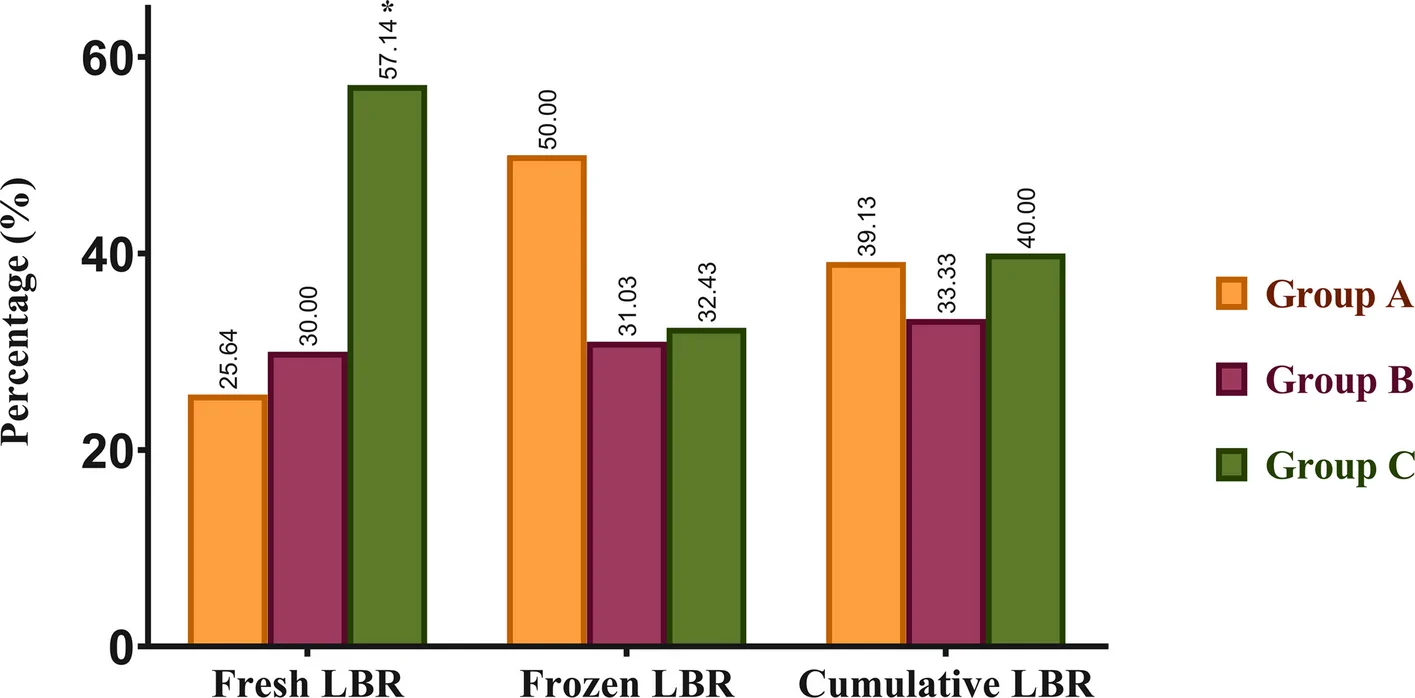
Live birth rate (LBR) in three groups. *Compared with Group A, *P* < 0.05

The live birth rates across the three groups were illustrated in Fig. 3. In fresh embryo transfer cycles, Group C had a significantly higher live birth rate (57.14%) compared to Group A (25.64%) (*P* = 0.04). In frozen embryo transfer cycles, no significant differences in live birth rate were observed among the groups (*P* > 0.05). The cumulative live birth rates were of no significant difference (39.13% in Group A, 33.33% in Group B, and 40.00% in Group C, respectively).

### Linear regression analysis of factors Influencing D3 good quality embryo rate

Table 3 presented the results of univariate and multivariate linear regression analyses to identify factors associated with the D3 good-quality embryo rate in different group classifications. Using Group A as the reference, univariate analysis showed that Group C had a significantly higher D3 good-quality embryo rate compared to Group A (β = 0.20, 95% CI: 0.10–0.30, *P* < 0.001). This association remained significant in the multivariate analysis after adjusting for ovarian stimulation protocol (β = 0.17, 95% CI: 0.05–0.28, *P* = 0.004). However, no significant differences were observed between Group B and Group A (*P* > 0.05). Using Group B as the reference, univariate analysis revealed that Group C had a significantly higher D3 good-quality embryo rate than Group B (β = 0.15, 95% CI: 0.05–0.26, *P* = 0.01). This association remained significant in the multivariate model after adjusting for confounders, including infertility duration and ovarian stimulation protocol (β = 0.12, 95% CI: 0.00–0.24, *P* = 0.04).

**Table 3:** Univariate and multivariate linear analysis of factors affecting D3 good-quality embryo rate

	Univariate linear analysis (β, 95%CI)	Multivariate linear analysis (β, 95%CI)	Univariate linear analysis (β, 95%CI)	Multivariate linear analysis (β, 95%CI)
Group A	Ref	Ref	/	/
Group B	0.05 (−0.05, 0.15)	0.04 (−0.06, 0.14)	Ref	Ref
Group C	0.20 (0.10–0.30)	0.17 (0.05, 0.28)	0.15 (0.05, 0.26)	0.12 (0.00, 0.24)

Variables with a *P*-value < 0.2 were included in the multivariate analysis as confounding factors. When using group A or group B as ref, the included confounding factors included age, infertility duration, primary infertility, basal FSH, basal LH, AFC, Gn duration, Gn dosage, ovarian stimulation protocol, fertilization type. Ovarian stimulation protocol was adjusted in multivariate analysis when using group A as reference. Infertility duration and ovarian stimulation protocol were adjusted in multivariate analysis when using group B as reference

*GH* growth hormone, *CI* confidence interval, *Ref* reference

## Discussion

In this study, we evaluated the effect of prolonged GH pretreatment on IVF outcomes in women with a previous cycle of poor quality embryos. Our findings demonstrate that prolonged GH pretreatment (12 weeks) is associated with improved embryo quality, as reflected by a higher D3 good-quality embryo rate compared with shorter-duration or no GH treatment.

In women with a history of poor quality embryos, improving embryo quality remains a persistent challenge. While some studies have investigated alternative approaches, such as modified culture systems, the effectiveness of these methods is still uncertain [24]. GH has been reported as a potential adjuvant therapy to improve embryo quality in IVF treatment for patients with poor embryo quality in previous cycles, yet studies on its efficacy have yielded inconsistent results [25–28]. A matched case–control study involving 1562 women showed that GH pretreatment (2 or 4 IU/d for 2–6 weeks) significantly increased the high-quality embryo rate [25]. However, a recent randomized controlled trial with 128 women found no significant improvement in high-quality embryo rates following 7 weeks of GH co-treatment (2–4 IU/d) [27]. Similarly, a retrospective cohort study reported no significant enhancement in good-quality embryo numbers with 6 weeks of GH pretreatment (2–5 IU/d) [28]. The discrepancies among these studies may be related to differences in patient selection and GH duration.

The development of follicles from the primordial follicle stage to the pre-ovulatory mature follicle typically takes about 85 days [20]. GH has been implicated in several key processes involved in oocyte maturation, including regulating energy metabolism, mitochondrial function, and intracellular signaling pathways, and reducing apoptosis [29]. In particular, GH is known to act through the GH/IGF-1 axis, which plays a crucial role in follicular development. GH can enhance granulosa cell proliferation and steroidogenesis, improve mitochondrial activity in oocytes, and promote cytoplasmic maturation through IGF-1-mediated signaling pathways [30]. Experimental studies have also shown that GH supplementation may improve oocyte competence by reducing oxidative stress and enhancing mitochondrial function, thereby supporting early embryonic development [31]. Since these processes occur throughout follicular growth rather than only during the final stage of ovarian stimulation, a longer duration of GH exposure may be necessary to exert a more complete effect on folliculogenesis and oocyte maturation. This may partly explain why prolonged GH pretreatment appeared to be more effective than shorter-duration treatment in our study. A recent study has shown that appropriately prolonging the use of GH can enhance the effect of GH on follicular development [32]. Interestingly, a study reported a case where GH replacement at a dose of 1.1 mg/d for 10 months normalized follicular fluid IGF-1 levels, improving oocyte and embryo quality and leading to a successful pregnancy [26]. Taken together, these findings provide preliminary biological and clinical support for the hypothesis that prolonged GH pretreatment may allow more comprehensive cytoplasmic and molecular improvement in oocytes, ultimately leading to better embryo quality.

Beyond embryo quality, Group C (12-week GH pretreatment) also showed a significantly higher live birth rate in fresh embryo transfer cycles (57.14%) in our study. This may be attributable to improved oocyte quality after GH pretreatment, as reflected by the higher D3 good-quality embryo rate and blastocyst formation rate, which may contribute to improved implantation potential. Additionally, the potential impact of GH on the endometrium has been reported to improve endometrium that is receptive to embryo implantation and thus increase the chances of a live birth in fresh cycles [33]. In contrast, no significant differences were observed in live birth rate after frozen embryo transfer or in cumulative live birth rate among the groups, likely due to freezing/thawing variables and embryo selection characteristics overshadowing GH effects. Moreover, the relatively small sample size in this study may have limited the statistical power, further contributing to the lack of significant differences.

An additional consideration is that the distribution of ovarian stimulation protocols differed among the three groups and may therefore have acted as a potential confounder. To address this issue, we performed both additional stratified analyses and multivariate regression analyses adjusting for stimulation protocols. The stratified analyses demonstrated a consistent trend toward higher D3 good-quality embryo rates with prolonged GH pretreatment across different protocol subgroups, without any contradictory patterns. Furthermore, multivariate analysis confirmed that prolonged GH pretreatment remained independently associated with improved D3 embryo quality after adjustment for ovarian stimulation protocol. Although most subgroup comparisons did not reach statistical significance, likely due to limited sample sizes, particularly in the PPOS subgroup, these findings collectively suggest that the beneficial effect of prolonged GH pretreatment is unlikely to be solely driven by differences in ovarian stimulation protocols.

The primary strength of this study lies in its real-world applicability, focusing on a well-defined patient population with specific inclusion criteria. All participants in our study were aged 38 years or younger, which provides a more homogenous group compared to other studies that included a broader age range. By focusing on a younger, more uniform age group, our study minimizes this potential confounding factor. Furthermore, our study employed stringent inclusion criteria to define poor-quality embryos, ensuring consistency in embryo selection. Specifically, good-quality embryos were defined as those with no fewer than six blastomeres, with equal or slightly unequal blastomere sizes, and less than 25% anucleate fragments. This precise definition of embryo quality enhances the reliability of our findings by limiting variability in embryo selection, providing a clearer understanding of the impact of GH treatment on embryo development.

Inevitably, several limitations should be considered. First, the retrospective design and the substantial attrition from 1,738 eligible women to 192 included participants may introduce inherent selection bias, which is difficult to eliminate in observational studies and may limit external validity. However, strict inclusion criteria were applied to ensure a highly homogeneous population with clearly defined poor embryo quality and age ≤ 38 years, which strengthens internal validity. Although the final cohort represents a selected subgroup, this design allows us to specifically address a clinically meaningful population of young patients with a history of poor embryo quality despite relatively preserved ovarian reserve. Second, the relatively small sample size may have limited the statistical power to detect subtle differences, particularly in subgroup analyses. Nevertheless, the direction of effect remained consistent across all analyses, supporting the robustness of the observed association. Third, the study did not include long-term follow-up data to assess the safety of GH treatment for both patients and their offspring. Although current published data has provided reassurance on the long-term safety profile of GH in patients receiving GH treatment [34], its long-term risks and benefits in the context of assisted reproduction remain unclear. Future prospective studies with larger sample sizes, long-term follow-up on obstetric outcomes, and comprehensive assessment of the long-term safety of GH use are essential to validate its clinical efficacy and ensure patient safety.

In conclusion, in women with poor embryo quality in previous IVF cycles, prolonged GH pretreatment for 12 weeks was associated with improved embryo quality and may be beneficial for embryo development. Although a higher live birth rate was observed in fresh embryo transfer cycles, no significant improvement was found in cumulative live birth rate. Further prospective studies are needed to clarify its effect on overall reproductive outcomes.

## Supplementary Information

Supplementary Material 1.

## Data Availability

The datasets used and/or analyzed during the current study are available from the corresponding author on reasonable request.
