# Supplementary material for: Prolonged growth hormone pretreatment is associated with improved embryo quality in women with poor quality embryos in previous IVF cycles
Source: BMC Pregnancy Childbirth. 2026 Aug 15;26:1311. doi: 10.1186/s12884-026-09825-4 (PMC13753138; doi:10.1186/s12884-026-09825-4)
Supplement: Supplementary file 1 [file 12884_2026_9825_MOESM1_ESM.pdf]

温州医科大学附属第一医院临床研究伦理委员会审查批件

（Review of Ethics Committee in Clinical Research (ECCR) of the First Affiliated Hospital of Wenzhou Medical University）

受理编号 Acceptance Number：KY2025-R021　　批件号：临床研究伦审 Issuing Number（2025）第（R021）号

项目名称<br>Project	生长激素预处理时长对既往胚胎质量差女性的 IVF 结局影响<br>(Effect of growth hormone pretreatment duration on IVF outcomes in women with poor previous embryo quality)		
申办者<br>Applicant	温州医科大学附属第一医院	试验目的<br>Objective	临床科研<br>Clinical research
试验科室<br>Department	生殖医学中心		
试验项目负责人<br>Principal Investigator	刘唱		
审查方式和时间<br>Form and Date	☐ 会议审查 Review Conference，时间：＿＿＿＿＿＿<br>☑ 快速审查 Fast track，时间：2025 年 1 月 15 日		
审查材料<br>Documents for Review	1、医学临床科研项目及伦理审查申请表，v1.0 版；<br>2、临床研究方案，v1.0 版，2025.1.6；<br>3、免除受试者知情同意书；<br>4、研究者团队成员目录（职责）；<br>5、主要研究者、团队成员简历及 GCP 证书，v1.0 版；<br>6、研究者责任声明；<br>7、CRF/临床观察表，v1.0 版。		
审查意见<br>Comments	根据国家卫健委《涉及人的生物医学研究伦理审查办法》(2023)、WMA《赫尔辛基宣言》和 CIOMS《人体生物医学研究国际道德指南》的伦理原则，经本伦理委员会审查，同意该项目开展。<br>According to the Regulations and Rules of "Ethical Reviews for Biomedical Research Involving Human Subjects" (2023)the National Health Commission of PRC , "Declaration of Helsinki" of WMA, and "International Ethical Guidelines for Human Biomedical Research" of CIOMS , the project was approved by ECCR.		
主任委员/副主任委员/受委托委员签字<br>Signature of the ECCR Chair		签发日期<br>Date	2025.1.18
温州医科大学附属第一医院临床研究伦理委员会（盖章）<br>Ethics Committee in Clinical Research of the First Affiliated Hospital of Wenzhou Medical University (Seal)			
附注（Note）：<br>1. 临床研究应在批准之日起 1 年内实施，逾期未实施，本批件自行废止。临床研究过程中将接受伦理委员会的			

温州医科大学附属第一医院 临床研究伦理委员会 专用章

CS 扫描全能王
3亿人都在用的扫描App

跟踪审查，审查频度为自批准之日起每12个月一次。（伦理委员会有权根据临床试验实际开展情况改变跟踪审查频度）
The clinical study shall be implemented within 1 year from the date of approval. If overdue, the approval for this project shall berevoked. During the implementation of clinical research, tracking review will be conducted by ECCRevery 12 months fromthe effective date of the initial approval (the ethics committee has the right to change the frequency of trackingreview according to the actual implementation of clinical trials)

2. 请严格遵从已批准的研究方案，如果方案修改需以书面形式报告伦理委员会，经伦理委员会批准后方可执行。Please strictly follow the approved research protocol. Any revisions of the protocol must be reported to ECCR in written form. It can be conducted only after the modification was approved by ECCR.
3. 发生严重不良事件以及影响研究风险受益比的非预期不良事件，须在24小时内报告本伦理委员会。Serious adverse events and unanticipated adverse events that affect the risk-to-benefit ratio of the project must be reported to ECCR within 24 hours.
4. 暂停、方案违背或提前终止临床研究，请及时上报本伦理委员会。Anysuspension, project violation or early termination of the clinical research, should be reported to ECCR promptly.
5. 完成临床研究，须提交研究完成报告给本伦理委员会。Please submit a completion research report to ECCR after completion of the project.
6. 经伦理审查委员会批准的研究在实施前，研究者需按国家医学研究登记备案信息系统要求分别如实、完整、准确上传，并根据研究进展及时更新信息。Before the implementation of research approved by the ECCR, researchers must truthfully, completely, and accurately upload the information according to the requirements of the National Medical Research Registration System (www.medicalresearch.org.cn), and update the information in a timely manner according to the research progress.
7. 涉及采集、保藏、利用、对外提供我国人类遗传资源，必须遵守《人类遗传资源管理条例实施细则》。To collect, preserve, utilize the Chinese human genetic resources, it is necessary to comply with the Implementation Rules of the Regulations on the Management of Human Genetic Resources.

伦理委员会声明：

★ 温州医科大学附属第一医院临床研究伦理委员会组成及工作程序遵循中国GCP、ICH-GCP及相关法律法规，其审查过程不受伦理委员会以外任何组织及个人影响。

★ 本伦理委员会各委员已签署保密协议，所有标准操作规程文件、机密信息、会议记录等及其副本的所有

权均归伦理委员会。

地址：浙江省温州市瓯海区南白象温州医科大学附属第一医院新院区　　邮编：325000
联系电话：0577-55578055　　传真：0577-55578033　　E-mail:wyyyclinical@126.com

扫描全能王
3亿人都在用的扫描App
